# A national retrospective survey of anisakidosis in France (2010-2014): decreasing incidence, female predominance, and emerging allergic potential

**DOI:** 10.1051/parasite/2018016

**Published:** 2018-04-11

**Authors:** Hélène Yera, Émilie Fréalle, Emmanuel Dutoit, Jean Dupouy-Camet

**Affiliations:** 1 Parasitology-Mycology Department, Cochin Hospital, Assistance Publique Hôpitaux de Paris, Paris Descartes University, 27 rue du Faubourg St Jacques, 75014 Paris France; 2 CHU Lille, Parasitology-Mycology Laboratory, 59000 Lille France; 3 Université de Lille, CNRS, Inserm, CHU Lille, Institut Pasteur de Lille, U1019–UMR 8204 − CIIL − Center for Infection and Immunity of Lille, 59000 Lille France

**Keywords:** Anisakidosis, Anisakiasis, human, Anisakis, incidence, allergy, fish

## Abstract

A retrospective survey was carried out over the years 2010–2014 among all Parasitology laboratories of University hospitals in France (ANOFEL network). The objective was to estimate the incidence of anisakidosis in France as new culinary habits such as the consumption of raw fish (sushi) or undercooked fish are increasing. A total of 37 cases of anisakidosis were notified by all French laboratories: 7 proven cases with evidence of a worm, 12 possible cases with abdominal pain after consumption of raw fish with detection of anti-*Anisakis* precipitins, and 18 allergic cases defined as acute manifestations after consumption of fish, associated with specific IgE for *Anisakis*. The median age of affected individuals was 42 years (11-69) and there was a significant predominance of women (67%). Compared with previous surveys in France, this study indicates a decrease in clinical cases of anisakidosis and illustrates the emerging allergic potential of anisakids.

## Abstract (fr)

Une enquête rétrospective a été réalisée au cours des années 2010-2014 auprès des laboratoires de parasitologie des hôpitaux universitaires de France (réseau ANOFEL). L’objectif était d’estimer l’incidence de l’anisakidose en France depuis l’émergence de nouvelles habitudes culinaires comme la consommation de poissons crus (sushi) ou insuffisamment cuits. Les laboratoires français de Parasitologie ont ainsi notifié 37 cas d’anisakidose: 7 cas certains avec présence du ver, 12 cas possibles se caractérisant par des douleurs abdominales après consommation de poisson cru et la présence de précipitines anti-*Anisakis* et 18 cas allergiques définis comme des manifestations aiguës après consommation de poisson associées à des IgE spécifiques anti-*Anisakis*. L’âge médian des cas était de 42 ans (11-69) et il y avait une prédominance féminine significative (67 %). Par rapport aux enquêtes précédentes en France, cette étude indique une diminution des cas cliniques d’anisakidose mais illustre le potentiel allergique émergent des anisakidés.

## Introduction

Anisakidosis is the infestation of humans by larvae of parasitic nematodes of the family Anisakidae whose adults live in the digestive tract of mammals such as cetaceans and pinnipeds [2,15,19,25]. Human infection occurs after ingestion of raw or poorly cooked fish. In humans, the larvae cannot develop in this unusual host (dead-end host), but their presence may result in acute symptoms (epigastric pain simulating gastric ulcer, caused by the fixation of a larva to the gastroduodenal mucosa), chronic symptoms (eosinophilic granuloma around a larva having penetrated the intestine and simulating a bowel tumor), or allergic symptoms (angioedema, acute recurrent or chronic urticaria, asthma, segmental bowel inflammation which may lead to obstruction, anaphylactic shock). The genera most often involved in human cases of infestation are *Anisakis* and *Pseudoterranova*. Most cases of anisakidosis are reported in Japan, Spain, the Netherlands and Germany [25]. The annual incidence in most European countries is around 20 cases/year [11]. In France, in 1989, Hubert *et al.* [16] reported an incidence of 8 cases/year (21 cases between January 1, 1985 and September 30, 1987) by surveying all Parasitology laboratories at University hospitals in mainland France. In 1995, Bourée *et al.* reported 25 cases that they had personally observed and identified 30 previously published cases [3]. Sporadic cases of anisakidosis are still regularly diagnosed but few are published [4,8,34]. Former studies had shown that the prevalence of infestation of marine fishes by anisakids was variable, but may be high [3,22,23]. These data were confirmed by the recent program “Fish-Parasites” (ANR-10-ALIA-004), which evaluated the prevalence of anisakids in the 15 most consumed fish species in France [26,31]. In this work, 1795 fishes were sampled between 2011 and 2013 during fishery research campaigns and from fish sellers inland. No anisakids were isolated from 44% of all fish sampled, whereas 34% of all fish had anisakids only in their viscera and 24% had anisakids either in their fillets only or in their fillets and viscera. The most parasitized fish species were saithe (*Pollachius virens*), megrim (*Lepidorhombus whiffiagonis*), cod (*Gadus morhua*), anglerfish (*Lophius piscatorius*), whiting (*Merlangius merlangius*) and hake (*Merluccius merluccius*). Most nematodes, identified by molecular methods, belonged to the genus *Anisakis*, mainly *A. simplex*, but *A. pegreffii*, *Pseudoterranova*, *Contracaecum* and *Hysterothylacium* were also identified.

In recent years, the growing popularity of consumption of raw fish such as sushi, carpaccio or prepared by multiple other recipes has been observed in France. The number of “Japanese” restaurants or fast-food shops serving and/or delivering sushi has increased dramatically. The purchase of prepared raw fish by French households increased by 230% (762 to 2540 tons) between 2005 and 2014 [13]. However, no recent data on the incidence of human anisakidosis are available in France. The objective of our study was to assess changes in annual incidence two decades after the first investigation published by Hubert *et al.* [16], using a similar methodology.

## Material and methods

The incidence of the disease over the period 2010–2014 was estimated by a national survey among all hospital Parasitology laboratories in mainland France (ANOFEL network). A questionnaire was sent to the 35 French hospital Parasitology laboratories in January 2015. Questions were “Did you diagnose cases of anisakidosis in the period 2010–2014” and, if so, what was its type “esophageal, gastroduodenal, allergic, eosinophilic granuloma or another form”? If the answer was positive, a more detailed questionnaire was sent to laboratories to collect epidemiological and bioclinical data, including the results and methodology for serological testing. The Laboratory of Lille University hospital receives requests for the detection of anti-*Anisakis* precipitins by immunoelectrophoresis (IEP) from a private medical laboratory having a national network and carried out, over the survey period, 566 serology assays. When anti-*Anisakis* detection by IEP was positive, prescribers were interviewed to obtain epidemiological and bioclinical information. These cases were included among the cases reported by all the other hospital Parasitology laboratories. All the cases were classified as follows: a confirmed case of anisakidosis was defined as the identification of a worm in a digestive sample; a possible case of anisakidosis was defined as any abdominal pain after raw fish consumption and the presence of anti-*Anisakis* precipitins; and an allergic case of anisakidosis was defined as the occurrence of acute allergic manifestations following fish consumption and associated with the presence of anti *Anisakis* IgE.

## Results

All the 35 Parasitology laboratories participated in the survey. Overall, 37 cases (Table 1) were identified and classified into 7 confirmed cases of anisakidosis (19%), 12 possible (32%) and 18 allergic cases (49%). The incidence of anisakidosis was 0.62 cases/month or 7.4 cases/year (37 cases over 60 months). Excluding the 18 cases of allergic anisakidosis, the incidence was 0.31 cases/month or 3.8 cases/year (19 cases over 60 months). When considering only confirmed cases, it was 0.1 cases/month or 1.4 cases/year. Among the confirmed cases, an esophageal form manifested as a few days of retro-sternal burning that stopped when the patient expelled a worm while vomiting. Five gastroduodenal forms were characterized by the observation of one (or several) worm(s) during gastric fibroscopy (with allergic swollen lips in one case). The 7^th^ confirmed case was a colonic form fortuitously diagnosed by the observation of larvae sections inside an eosinophilic granuloma removed during colonoscopy. In possible cases of anisakidosis, patients had epigastric or abdominal pain after raw fish consumption and assays of anti-*Anisakis* precipitins by IEP were positive. Cases of allergic anisakidosis were characterized by various cutaneous manifestations (pruritus, urticaria, swollen lips or swelling of the face) or by general malaise and, in one case, by anaphylactic shock. These forms were sometimes associated with acute epigastric or abdominal pain. The median age of affected individuals was 42 years (11–69) and there was a significant predominance of women (25 women versus 12 men, chi^2^ *p* < 0.05). The same predominance, though not significantly different, was also observed in allergic cases (13 women versus 5 men). Regardless of the clinical form, anti-*Anisakis* specific IgE were always detected when they were tested for, even in the absence of patent allergic manifestations (2 of 3 confirmed cases, 3 of 3 possible cases, and 18 of 18 allergic cases). Blood hypereosinophilia was sometimes mentioned but these data were not systematically evaluated. Confirmed cases of anisakidosis were treated by removal of the larvae during fibroscopy (5 cases) or with albendazole (2 cases). Possible cases were sometimes treated with albendazole (2 cases) or ivermectin (1 case). Antihistamines and local or systemic corticosteroids were prescribed to treat allergic cases (5 cases), as well as albendazole (5 cases). When the data were available, fish incriminated by patients were salmon (8 cases), anchovies (5 cases), tuna and mackerel (4 cases for each species), cod and herring (3 cases for each), sea bream and hake (2 cases each), and other fish (3 cases). The distribution of fish species according to the different forms of anisakidosis is given in Table 2. Finally, the geographic distribution of cases showed that 35% of cases were observed in Paris and suburbs, 35% in other regions and only 29% in coastal regions.

**Table 1:** Number of cases of anisakidosis (confirmed, possible or allergic) identified in France, 2010–2014.

	2010	2011	2012	2013	2014	total
Confirmed	1	1	2	1	2	7
Possible	1	1	2	2	6	12
Allergic	2	1	2	8	5	18
Total	4	3	6	11	13	37

**Table 2:** Fish incriminated in the occurrence of cases of anisakidosis identified in France, 2010–2014. For some cases, the species was unknown. For one case several species were possible.

	Confirmed	Possible	Allergic	Total
salmon	1	1	6	8
anchovies	1	2	2	5
mackerel	1		3	4
tuna		2	2	4
cod	1	1	1	3
herring	1		2	3
hake			2	2
sea bream	1	1		2
others	1	1	1	3

## Discussion

Our study is the first to estimate the incidence of anisakidosis in France since the survey by Hubert *et al.* published in 1989 [16], who identified 21 cases over a nearly 3-year period, including 10 confirmed anisakidosis cases and 11 patients with positive serology and having abdominal pain after eating raw fish, which could be considered as possible cases. No cases of allergic anisakidosis were identified since this clinical form was not known in 1989 and specific anti-*Anisakis* IgE assays were not available. In our study, over a 5 year-period (2010–2014), 37 cases were observed. Excluding the cases of allergic anisakidosis, we identified 19 cases over 60 months (0.31 cases/month), while 21 cases over 33 months (0.63 cases/month) were identified by Hubert *et al.* in 1989 [16]. When considering only confirmed cases, the monthly incidence of anisakidosis was 0.3/month in the former survey compared to 0.1 cases/month in our study. Consequently, our survey demonstrates a decrease in the incidence of parasitological cases over the past 25 years. Conversely, we found that the allergic potential of anisakids was emerging. This emergence was also confirmed by the analysis of data from the French National Allergy-Vigilance Network which reported, during the period 2010–2014, six severe cases of allergy to anisakids, whereas no cases were reported in the 2001–2008 period [9]. The emergence of allergy to anisakids has also been reported in international studies indicating an association between allergy to *Anisakis* and urticaria, or other allergic manifestations [2,6,12,22,25]. These aspects are well known in Spain or in Italy [1,21]. In France, allergic manifestations to anisakids have been reported [10,22], but their precise incidences still need to be evaluated. The analysis of a nationwide hospital medical information database (PMSI) database identified, between 1997 and 1999, 19 cases of anisakidosis for which the disease was recorded as the main reason for hospitalization [14]. The analysis of the same database (PMSI) during the period 2010–2014 identified 19 hospitalized cases for which anisakidosis was reported as the main diagnosis of hospitalization [9]. Therefore, the incidence of anisakidosis had decreased between these two periods: an average of 6.3 cases/year in the period 1997–1999 vs 3.8 cases/year in the period 2010–2014. This tendency to a decrease in cases had already been found by Petithory in 2007 [22]. This author identified 25 cases between 1977 and 1991 (2 cases/year) and only 6 cases between 1992 and 2005 (1 case every 2 years). A European Directive of 1991 requiring deep-freezing of fish sold to be consumed raw was suggested as the reason underlying the decreasing incidence. The low incidence of human disease contrasts with the very high prevalence of anisakids found in fish and the recent modifications of culinary habits leading to higher consumption of raw fish. The high prevalence sometimes reported in fish directly examined at sea or bought from fishermen reflects the natural status of infection of fish, but not necessarily consumer exposure. Several causes may explain this discrepancy. At the level of sea professionals, anisakids are a major concern because they can lead to removal from sale and destruction of heavily parasitized batches. When preparing fillets, fishmongers often work on candling-tables and are able to cut the sides and, thus, eliminate the most patent parasites. European health regulations are also very strict and precise. They prohibit the marketing of fish intended to be eaten raw for catering if it has not been cured by appropriate measures such as freezing [19,24]. In addition, many sushis are prepared with farmed fish which, in general, are not parasitized [17]. Specialized television programs have promoted preventive methods (such as freezing) which are also popularized on the internet. However, it is uncertain whether these methods are used by individual consumers. Finally, concerning allergies, freezing or heating does not avoid allergy to anisakids as some allergens are thermo-tolerant [23,30,32]. The fish species incriminated in the occurrence of anisakidosis cases were salmon (8 cases), anchovies (5 cases), mackerel, and tuna (4 cases each). Although the latter are usual sources of infection by anisakids, the frequent incrimination of salmon was rather surprising as most salmon consumed in France are Atlantic farmed salmon which are usually not parasitized [17]. Wild Pacific salmon of the genus *Oncorhynchus* are also consumed in France and were sources of diphyllobothriosis [33]. These Pacific salmon are known to host anisakids; Setyobudi *et al.* examined 120 Pacific salmon and found that all were parasitized with a high level of anisakid larvae, mostly found in muscles [27]. In our survey, we were not able to obtain more details on the origin of the salmon. Analysis of the geographical distribution of cases does not show any regional predominance. We are not aware of different regional culinary habits concerning fish preparations in France, and most fish are distributed and sold all over the country by several networks of supermarkets. Therefore, parasitized fish can be found everywhere. We did not find the marked geographic differences found in Italy, where sensitization rates showed marked geographic differences (range: 0.4-12.7%), being highest along the Adriatic and Tyrrhenian coasts, where homemade marinated anchovies are an age-old tradition. In inland centers in northern Italy, the prevalence was directly related to the number of inhabitants. Interestingly, the analysis of the impact of immigration on the prevalence of *Anisakis* hypersensitivity showed that about 60% of sensitized subjects in Milan and Turin came from southern Italy or from non-European countries. [1]. Our survey also showed a significant predominance of cases in women. This female predominance was not known and was not found in the study of Hubert *et al.* [16]. Women may consume some pieces of raw fish during the domestic preparation of fish dishes and consequently be more exposed to the parasite than men. There are no data showing that French women consume more raw fish than men. A 2006 Japanese study showed that older women were the heaviest sushi consumers [7]. However, such differences were not found in an analysis of a recent population survey carried out in the United States to examine differences in the consumption of various types of foods between men and women [28]. A predominance of anisakidosis in women is mentioned by Sohn *et al.* [29] in Korea (45.8% men versus 54.2% women). Interestingly, sex hormones have been reported to play a possible role in the homeostasis of immunity as women could be more susceptible to allergic diseases [5] and this could explain the slight predominance of women having allergic forms in our study. However, our study has some limitations. There are certainly many asymptomatic cases, or cases involving people with very few symptoms who do not consult physicians for their illness. Furthermore, confirmed cases can be diagnosed only by the observation of the larva, which limits this diagnosis to departments practicing endoscopy. Our study shows the emergence of allergy to the anisakids. However, the detection of *Anisakis simplex*-specific IgE is questionable because multiple cross reactions, through the allergen Ani s 3 (tropomyosin), can be observed between anisakids and mites, crustaceans, and molluscs. The detection of IgE directed against a very specific antigenic fraction (Ani s 1) is available in the expensive multi-testImmunoCAP^®^ ISAC [18]. Recent data have shown a strong correlation between the results of this test and those from western blot analysis of ES or crude antigens [20].

In conclusion, our survey shows that in France half of the cases of human anisakidosis are allergic forms; the other half is divided into confirmed and possible cases. We also showed a predominance of cases in women. Sometimes, although this could not be precisely quantified, clinical forms may be combined: confirmed cases with allergic manifestations, possible cases sensitized to anisakids and allergic cases with epigastric pain a few hours after consumption of raw fish. It is not possible to assess the level of underestimation in the total number of cases because some uncertainties remain: the number of asymptomatic cases, the number of patients who do not consult a physician, the number of specific analyses prescribed to confirm anisakidosis when patients are seen in medical departments, and the number of allergic forms without digestive symptoms which are potentially misclassified as fish allergy. For these reasons, a better evaluation of the risk of anisakids for public health is needed in France.

## Conflict of interest

The authors declare that they have no conflicts of interest in relation to this article.

## References

[1] AAITO-IFIACI *Anisakis* Consortium. 2011. *Anisakis* hypersensitivity in Italy: prevalence and clinical features: a multicenter study. Allergy, 66, 1563-1569. 10.1111/j.1398-9995.2011.02691.x 21851362

[2] Audicana, MT, Kennedy, MW. 2008 *Anisakis simplex*: from obscure infectious worm to inducer of immune hypersensitivity. Clinical Microbiological Review, 21, 360-379. 10.1128/CMR.00012-07 PMC2292572 18400801

[3] Bourée, P., Paugam, A., Petithory, J.C. 1995 Anisakidosis: report of 25 cases and review of the literature. Comparative Immunology, Microbiology and Infectious Diseases, 18, 75-84. 10.1016/0147-9571(95)98848-c 7621671

[4] Brunet J, Pesson B, Royant M, Lemoine JP, Pfaff AW, Abou-Bacar A, Yera H, Fréalle E, Dupouy-Camet J, Merino-Espinosa G, Gómez-Mateos M, Martin-Sanchez J, Candolfi E. 2017, Molecular diagnosis of *Pseudoterranova decipiens s.s* in human, France. BMC Infectious Diseases, 6, 397. 10.1186/s12879-017-2493-7 PMC5460327 28583155

[5] Chen W, Mempel M, Schober W, Behrendt H, Ring J. 2008 Gender difference, sex hormones, and immediate type hypersensitivity reactions. Allergy, 63, 1418-1427. 18925878 10.1111/j.1398-9995.2008.01880.x

[6] Daschner A, Pascual CY. 2005 *Anisakis simplex*: sensitization and clinical allergy. Current Opinion in Allergy and Clinical Immunology, 5, 281-285. 15864089 10.1097/01.all.0000168795.12701.fd

[7] De Silva D, Yamao M. 2006 A yen for sushi: an analysis of demographic and behavioral patterns of sushi consumption in Japan. Journal of Foodservice, 17, 63-76.

[8] Dupouy-Camet J, Gay M, Bourgau O, Nouchi A, Léger E, Dei-Cas E. 2014 L’atteinte œsophagienne : une complication rare de l’anisakidose à *Pseudoterranova*. Presse Médicale, 43, 81-83. 10.1016/j.lpm.2013.01.070 23727011

[9] Dupouy-Camet J, Touabet-Azouzi N, Fréalle E, Van Cauteren D, Yera H, Moneret-Vautrin A. Incidence de l’anisakidose en France. Enquête rétrospective 2010-2014. 2016. Bulletin Epidémiologique Hebdomadaire, 5-6. 64-70. Retrieved 8 January 2018 from http://www.invs.sante.fr/beh/2016/5-6/2016_5-6_1.html

[10] Eldin de Pécoulas P, Paugam A., Bourée P. 2014 Anisakiose et allergie : une association morbide négligée ? Revue Francophone des Laboratoires, 464, 89-95.

[11] European Food Safety Authority. 2010. Scientific opinion on risk assessment of parasites in fishery products. EFSA panel on biologic hazards (BIOHAZ). EFSA Journal, 8, 1543.

[12] Falcão H, Lunet N, Neves E, Iglésias I, Barros H. 2008 *Anisakis simplex* as a risk factor for relapsing acute urticaria: a case-control study. Journal of Epidemiology and Community Health, 62, 634-637. 18559447 10.1136/jech.2007.061572

[13] FranceAgriMer, 2015. Consommation des produits de la pêche et de l’aquaculture, 2014. pp.28 Retrieved 8th of January 2018 from http://www.franceagrimer.fr/content/download/38313/352902/file/STA-MER-CONSO%202014-mai2015.pdf

[14] INVS. 2004. Morbidité et mortalité dues aux maladies infectieuses d’origine alimentaire en France Institut de Veille Sanitaire St Maurice, France Retrieved 8^th^ of January 2018 from http://invs.santepubliquefrance.fr/publications/2004/inf_origine_alimentaire/inf_origine_alimentaire.pdf.

[15] Hochberg NS, Hamer DH, 2010 Anisakidosis: Perils of the deep. Clinical Infectious Diseases, 51, 806-812 20804423 10.1086/656238

[16] Hubert B, Bacou J, Belveze H. 1989 Epidemiology of human anisakiasis: incidence and sources in France. American Journal of Tropical Medicine and Hygiene, 40, 301-303. 2929852 10.4269/ajtmh.1989.40.301

[17] Lunestad BT. 2003 Absence of nematodes in farmed Atlantic salmon (*Salmo salar* L.) in Norway. Journal of Food Protection. 66, 122-124. 12540192 10.4315/0362-028x-66.1.122

[18] Martínez-Aranguren RM, Gamboa PM, García-Lirio E, Asturias J, Goikoetxea MJ, Sanz ML, 2014 *In vivo* and *in vitro* testing with rAni s 1 can facilitate diagnosis of *Anisakis* *simplex* allergy. Journal of Investigational Allergology and Clinical Immunology, 24, 431-438. 25668895

[19] Mattiucci S & D’Amelio S. 2014. Anisakiasis, in Helminth Infections and their Impact on Global Public Health, Bruschi F, Editor. Springer-Verlag: Wien. P., 325-365.

[20] Mattiucci S, Colantoni A, Crisafi B, Mori-Ubaldini F, Caponi L, Fazii P, Nascetti G, Bruschi F. 2017 IgE sensitization to *Anisakis pegreffii* in Italy: Comparison of two methods for the diagnosis of allergic anisakiasis. Parasite Immunology, 39 (7). DOI: 10.1111/pim.12440. 28475216

[21] Moro Moro, M., Tejedor Alonso, M.A., Esteban Hernández, J, Múgica García M.V., Rosado Ingelmo, A., Vila Albelda, C., 2011 Incidence of anaphylaxis and subtypes of anaphylaxis in a general hospital emergency department. Journal of Investigational Allergology and Clinical Immunology, 21, 142-149. 21462805

[22] Petithory JC. 2007 Données nouvelles sur l’anisakidose. Bulletin de l’Académie Nationale de Médecine (Paris), 191, 53-65. 17645107

[23] Petithory JC. 2008 Actualités sur l’anisakidose. Revue Française des Laboratoires, 399, 87-93.

[24] Pozio E. 2013 Integrating animal health surveillance and food safety: the example of *Anisakis*. Scientific and Technical Review of the Office International des Epizooties (Paris), 32, 487-496. 10.20506/rst.32.2.2246 24547652

[25] Pravettoni V, Primavesi L, Piantanida M. 2012 *Anisakis simplex*: current knowledge. European Annals of Allergy and Clinical Immunology, 44, 150-156. 23092000

[26] Seesao Y. 2015. Caractérisation des Anisakidae dans les poissons marins : développement d’une méthode d’identification par séquençage à haut-débit et étude de prévalence. Thèse de doctorat en Biologie. Université Lille 2.

[27] Setyobudi E, Jeon CH, Lee CH, Seong KB, Kim JH. 2011 Occurrence and identification of *Anisakis* spp. (Nematoda: Anisakidae) isolated from chum salmon (*Oncorhynchus keta*) in Korea. Parasitology Research. 108, 585-592. 20938685 10.1007/s00436-010-2101-x

[28] Shiferaw B, Verrill L, Booth H, Zansky SM, Norton DM, Crim S, Henao OL. 2012 Sex-based differences in food consumption: Foodborne Diseases Active Surveillance Network (FoodNet) Population Survey, 2006-2007. Clinical Infectious Disease, 54 Suppl 5, S453-S457. 10.1093/cid/cis247 22572669

[29] Sohn WM, Na BK, Kim TH, Park TJ. 2015 Anisakiasis: Report of 15 gastric cases caused by *Anisakis* Type I larvae and a brief review of Korean anisakiasis cases. Korean Journal of Parasitology, 53, 465-470. 26323845 10.3347/kjp.2015.53.4.465 PMC4566497

[30] Ventura MT, Tummolo RA, Di Leo E, D’Ersasmo M, Arsieni A. 2008 Immediate and cell-mediated reactions in parasitic infections by *Anisakis simplex*. Journal of Investigational Allergology and Clinical Immunology 18(4), 253-259. 18714532

[31] Verrez-Bagnis V, Seesao Y, Thebault A, Gay M, Aliouat-Denis CM, Le Fur B, Cuzzucoli D, Cos I, Jérôme, M, Kolypczuk L, Bruzac S, Audebert C, Dei-Cas E, Viscogliosi E. and the “Fish-Parasites” consortium. 2015. Prevalence of nematodes (Anisakidae) in fish species most consumed in France. Abstract book of the 5th Trans-Atlantic Fisheries Technology conference, Nantes, France. Retrieved 8 January 2018 from http://wwz.ifremer.fr/taft2015/content/download/90381/1110052/file/book%20of%20abstracts%20final.pdf

[32] Vidacek S, de las Heras C, Solas MT, Mendizábal A, Rodriguez-Mahillo AI, Tejada M. 2010. Antigenicity and viability of *Anisakis* larvae infesting hake heated at different time-temperature conditions. Journal of Food Protection, 73, 62-68. 10.4315/0362-028x-73.1.62 20051205

[33] Yera H, Estran C, Delaunay P, Gari-Toussaint M, Dupouy-Camet J, Marty P. 2006. Putative *Diphyllobothrium nihonkaiense* acquired from a Pacific salmon (*Oncorhynchus keta*) eaten in France; genomic identification and case report. Parasitology International. 55, 45-49. 10.1016/j.parint.2005.09.004 16243582

[34] Yera H, Fréalle E, Dupouy-Camet J. 2016. Molecular confirmation of *Anisakis pegreffii* as a causative agent of anisakidosis in France. Digestive and Liver Disease, 48, 970. 10.1016/j.dld.2016.04.003 27133969

